# The Feasibility of Using Pulsatile Electromagnetic Fields (PEMFs) to Enhance the Regenerative Ability of Dermal Biomaterial Scaffolds

**DOI:** 10.3390/jfb9040066

**Published:** 2018-11-19

**Authors:** Dale S. Feldman

**Affiliations:** Department of Biomedical Engineering, University of Alabama at Birmingham, Birmingham, AL 35294, USA; dfeldman@uab.edu; Tel.: +1-(205)-807-2445

**Keywords:** electrical stimulation, skin wound healing, adjunctive wound healing therapy

## Abstract

Degradable regenerative scaffolds usually require adjunctive treatment to meet the clinical healing performance requirements. This study was designed to look at pulsatile electromagnetic fields (PEMF) as an adjunctive therapy for these scaffolds in skin wounds; however, no scaffold was used in this study in order to isolate the effects of PEMF alone. In this study, New Zealand rabbits received four full-thickness defects with a size of 3 cm × 3 cm on the dorsolateral aspect. The rabbits in the treatment group were placed in a chamber and subjected to a PEMF at six different predetermined frequency and intensity combinations for 2 h a day for a 2-week period. At the end of the 2-week period, the animals were sacrificed and tissue samples were taken. Half of each tissue sample was used for histomorphometric analysis and the other half was for tensile testing. The study showed an increased healing response by all the PEMF treatments compared to that in the control, although different combinations led to increases in different aspects of the healing response. This suggests that although some treatments are better for the critical clinical parameter—healing rate, it might be beneficial to use treatments in the early stages to increase angiogenesis before the treatment is switched to the one best for the healing rate to get an even better haling rate.

## 1. Introduction

Skin injuries are one of the chief causes of death in North America for people between the ages of 1 and 44 [1]. The two major populations in the U.S. are skin ulcers (7 million patients/year) and burns (2 million patients/year) [2,3].

Current non-surgical treatment for skin wounds is usually limited to passive modalities such as debridement, cleansing, and use of inert dressings. The problem is the cost for the patient (both medical and lost wages) due to the long time required for these skin wounds to heal. Surgery is used in many cases to shorten the healing time, but is a costly option. Approved active treatments tend to also be costly and only are currently approved for a few clinical applications [4,5]. In addition, these systems have only shown moderate cost-effectiveness.

For example, pressure ulcers in spinal cord-injured (SCI) patients are a problem since they occur in 20%–30% of these patients [6,7,8]. In the U.S., there are about 2.5 million patients with pressure ulcers per year at a cost of about $10 billion/year, with a pressure ulcer increasing the average cost of just a hospital stay by over $40,000 [9]. About 60,000 patients die each year due to complications of pressure ulcers [9].

There are currently two main treatment options for (Stages III and IV) SCI pressure ulcer patients: non-surgical treatment requiring roughly six months of bed rest and continual dressing changes or surgical treatment (most common skin flap surgery) requiring roughly six weeks of bed rest [10]. Though surgical treatment typically produces faster healing, there is still a significant amount of bed rest required, and recurrence rates are high [10].

Surgical treatment is also costly, adding over $40,000 to total costs [11]. Non-surgical treatments, however, with additional bed rest, can have significantly more costs of $6000/day in-patient and up to half of that at home [12].

Therefore, there is a need to develop a non-surgical treatment for pressure ulcers that is more comparable to surgery in terms of cost and bed rest time. A reasonable goal is to double the healing rate of a non-surgical treatment to approximate the bed rest time required for graft surgery in the SCI population. A number of biomaterial scaffolds have been under investigation, but they have not been able to approach this design constraint without added biologics (e.g., stem cells and growth factors) [5,6,13]. The use of these added biologics can dramatically increase the development time and cost in part due to the additional regulatory hurdles for these systems.

This study aims to look at the feasibility of using electrical stimulation instead of these added biologics to enhance the regenerative ability of biomaterial scaffolds. This study is looking for the range of bioactivity the electrical stimulation can impart without a biomaterial present, which will be added in future studies.

In the 1960s, Becker [14] developed the concept of “current of injury”, in which an injury causes a localized shift in current flow. Although the exact mechanism is still unclear, this current flowing through biological tissue elicits electrochemical, electrophysical and electrothermal effects on the tissue and cells, which can enhance healing [15]. This electric field has been shown to stimulate cellular function, amino acid uptake, ATP resynthesis, protein synthesis [16], mitosis [17], receptor expression [18], and motility [19]. 

The benefit of electrical stimulation can be that it both mimics the normal “current of injury” when it is absent, as well as amplifying it when it is present. Evidence suggests that supplementation of this current can stimulate a non-healing or chronic wound to heal as well as speeding the healing in an acute wound [20,21,22,23,24,25]. In a meta-analysis of several clinical studies [26], it was found that electrical stimulation resulted in a 13% improvement in the percentage of healing per week for all study designs included.

There are different types of electrical stimulation. Direct current (DC) stimulation has been shown to attract inflammatory cells to a wound site [27] and accelerate healing rates [20]. Clinically, electrical stimulation has been able to heal chronic leg ulcers that had resisted conventional treatment for several years [22,23,24,25,26,27,28]. Studies have consistently shown the treated group has a healing rate 1.5 to 2.5 times faster than that for the controls, as well as an antimicrobial effect, a decrease in pain, and more flexible scar tissue [23,24,25]. 

Investigators have also found success with pulsed or alternating current (AC) systems at both low and high voltages [29,30]. A Dermapulse^®^ system delivers pulsed galvanic currents to a wound. The treatment usually consists of 30-min sessions twice daily with 128 pulses per second and an amplitude of 35 mA. In a double blind-randomized placebo-controlled treatment, the Dermapulse^®^ healed [30] ulcers at twice the rate for the sham group. Further, in the crossover study, treated patients healed four times as fast as the untreated patients.

A few studies have looked at alternating current or asymmetric biphasic stimulation modalities and compared them to DC stimulation [31,32,33,34,35,36]. In general, DC stimulation seemed to enhance tissue perfusion and decrease the wound area more significantly than AC [34], but AC stimulation, however, reduced wound volume more than DC [35]. 

Pulsatile electromagnetic fields (PEMFs) that stimulate local electric fields have also been used [37,38,39,40]. This is an attractive option, since it is the only way to provide the electrical stimulation without contacting the wound. The PEMF was first used clinically for long-bone non-union fractures. During treatment, several surgeons reported increased healing of chronic skin ulcers near the treatment regime [41,42]. Many studies [38,39,40,41,42,43,44,45] have been done over the years, using PEMF, but have used a variety of frequency and intensity levels. Little has been done to determine if different combinations of frequencies and intensities alter the healing response and if there are optimum PEMF regimes. The goal of this study, therefore, was to evaluate the effects of PEMF at different combinations of frequencies and intensities. If, as expected, different PEMF treatments induce different types of bioactivity, it should be possible to select the optimal PEMF for a given stage of regenerative healing of the scaffold, even tailoring it to how the healing is progressing in a given clinical case. No scaffolds, however, were used in this study in order to determine the effects of PEMF apart from a specific scaffold. It is likely that the interaction of PEMF with different scaffolds will be different.

## 2. Materials and Methods

### 2.1. Materials

The PEMF system consisted of a generator and a rabbit chamber. The generator is an EBI Advanced Necrosis Treatment System model 300 (Electro-Biology, Incorporated, Parsippany, NJ, USA) that was able to supply power to the induction coils at frequencies of 65, 70, 75, 80, and 85 Hz and a pulse width of 0.38 msec. The induction coils were also capable of producing intensity levels of 3.3, 6.6, and 9.9 Gauss (corresponding to 0.33. 0.66, and 0.99 Millitesla, respectively). 

The induction (Helmholtz) coils were attached to the side of a fiberglass chamber with Velcro strips. These coils could be adjusted so that the center point of the coil was in the center of the wound area receiving the stimulation. The chamber was also designed to restrain the animal during treatment. 

### 2.2. Methods

**Figure 1:**
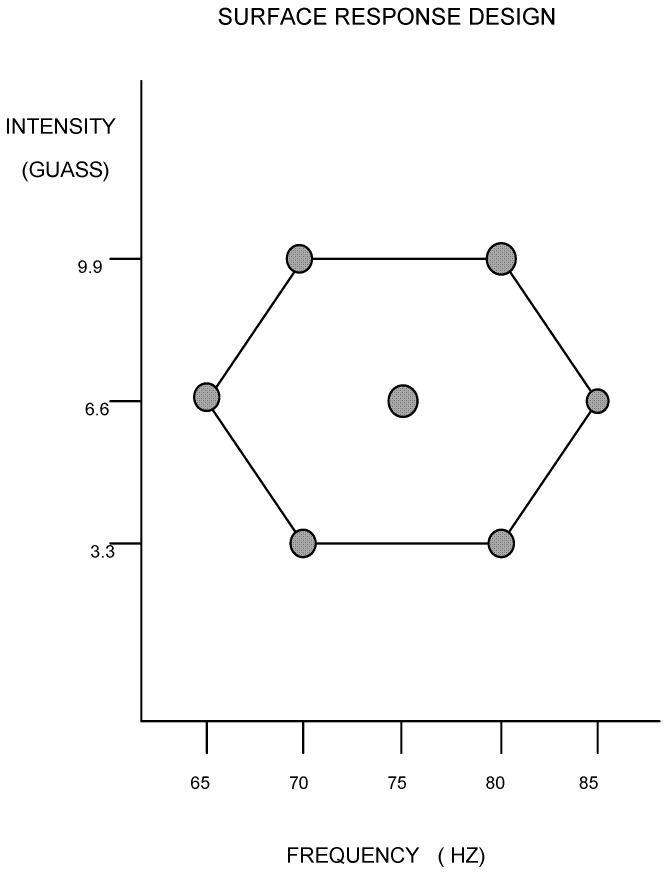
Hexagon response surface design.

Sixteen white New Zealand rabbits were divided into eight groups of two rabbits each: one control group and seven treatment groups. A response surface design was used to determine the various treatment combinations (Figure 1). This design allowed for each parameter (cellular tissue volume fraction, healing rate, and mechanical strength) to later be plotted as a contour map where the maximum or minimum points could be determined.

**Figure 2:**
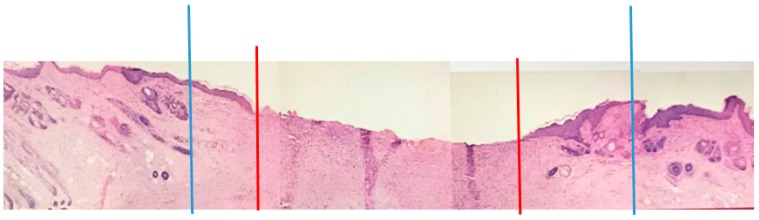
A representative cross-section of the wound after one week. The tissue removed is between the two blue lines down to the muscle (not shown). All the tissues between the blue lines formed during the first week. Between the blue and red lines on each side is the newly formed epidermis growing over the new dermis.

One day prior to surgery, the surgical area of each animal was shaved and a depilatory agent (Bikini Bare, Lee Pharmaceutical, El Monte, CA, USA) was used to thoroughly remove all hair. On the day of surgery, the animals were anesthetized using Ketamine/Ace Promazine (Aveco, Fort Dodge, IA, USA) and scrubbed using Betadine surgical Scrub (Purdue Fredric Co., Norwalk, CT, USA). Four full-thickness defects with a size of 3 cm × 3 cm down to the cutaneous trunci muscle were made on the dorsolateral aspects of the rabbit (Figure 2). Figure 2 is a cross-section of the wound after 1 week of healing.

The wound was then traced with sterilized tracing paper. Each wound was covered with an oxygen permeable dressing (Tegraderm^®^, 3M, Minneapolis, MN, USA) that was secured with surgical tape. An orthopedic stockinette (Johnson & Johnson) was then styled to fit the rabbit, which was covered by a mesh jacket (Alice King Chatham Medical Arts, Los Angeles, CA, USA) to prevent the rabbit from agitating the wound.

Each animal in the treatment groups was placed in the chamber and treated with the PEMF at various combinations of frequencies and intensities for 2 h a day for 10 days during a 2-week period. The control animals were also placed in the same chamber for the same time period without any PEMF. After 2 weeks, the animals were sacrificed using an overdose of Uthol and tissue samples were harvested. The harvested tissue specimens were cut in half with one piece placed in saline to be used for mechanical studies while the other was placed in an alcohol fixative for light microscope studies. 

Tensile testing was performed on the strip of tissue (approximately 5 mm wide and 3 mm thick) within 4 h of necropsy. Balsa wood pieces were glued using a cyanoacrylate adhesive to the ends of the tissue, leaving the central healed portion of the wound exposed, to reduce slippage in the mechanical grips. The tensile test was performed at a cross-head speed of 2.54 cm/s, producing a force–displacement curve from the relaxed state until separation. 

For histology, the samples were embedded in paraffin, cut at 5 μm thickness and stained with either a modified trichrome stain or hematoxylin and eosin stain. For each wound, the epithelialization (ER) and contraction (CR) rates (mm/week), along with volume fractions of neutrophils, macrophages, fibroblast, collagen, and blood vessels (BV), were determined. In addition, the volume fraction of cells, BV, and collagen were determined from sections of normal skin taken from two rabbits in one of the treated group (75, 6.6), which is in the center of the hexagon.

The volume fractions of the neutrophils, macrophages, and fibroblast were evaluated by histomorphometric techniques with a 121-point grid [46]. Ten placements near the center of the wound and near the edge of the wound were used for each wound. The volume fraction of the collagen was evaluated by color separation using the trichrome stain with an IBAS image analysis system (Kontron Imaging Systems, Munich, Germany). For the BV evaluation, the BV in a trichrome section were outlined. The image analysis program determined the volume fraction of BV and the average BV diameter.

The ER and CR rates were determined from histological measurements. The percentage of ER (EPI) was calculated by:

EPI = EL/WL × 100%

where EL is the ER length over the wound and WL is the wound length. The rate of ER in mm/week was calculated by:

ER = EL/(2 sides × 2 weeks).

The percentage of CR of the wound (CON) was determined by:

CON = [1 − (WL/WLO)] × 100%(1)

where WLO is the original wound length at the time of surgery (3 cm). The rate of CR, in mm/week, was determined by:

CR = (WLO − WL)/(2 sides × 2 weeks).(2)

The overall wound closure rate (WCR) was ER + CR for a given wound.

Statistics were done using the SAS statistics software program (SAS Institute, Inc. Cary, NC, USA). Using the surface response design method of statistics included in the General Linear Model (GLM) procedure, a three-dimensional regression was constructed on the responses measured at the specified points from a two-dimensional grid. A two-tailed Student’s *t*-test was used to determine statistical difference between the groups at *p* < 0.05.

## 3. Results

**Figure 3:**
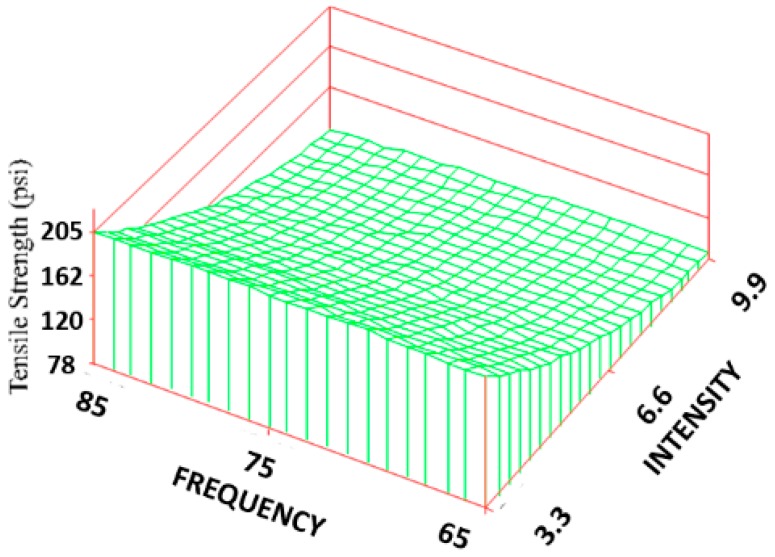
Surface response regarding tensile strength. The regression equation is: TS = 78.21 + 7.21(F) − 78.8(I) − 0.04(F^2^) + 4.2(I^2^) − 0.2(F)(I), where F is the designating frequency and I is the designating intensity.

**Figure 4:**
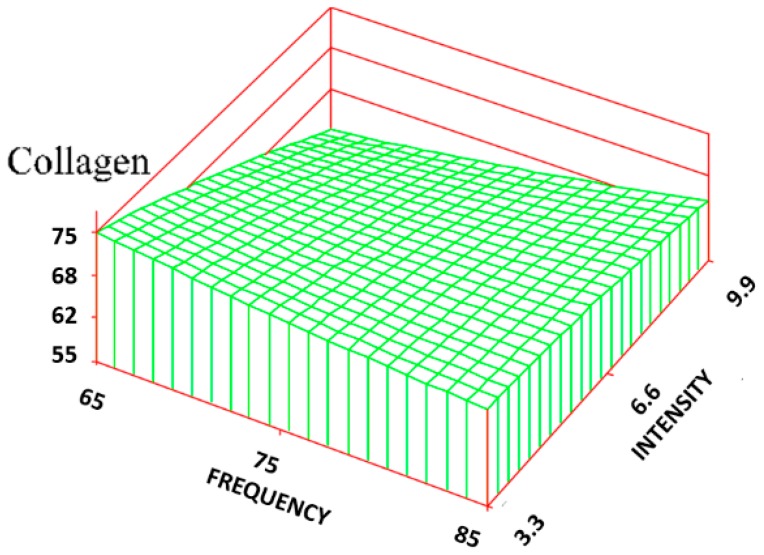
Surface response regarding the volume fraction of collagen. The regression equation is: C_V_ = 62.9 − 0.33(F) + 7.68(I) − 0.01(F^2^) − 0.09(I^2^).

**Figure 5:**
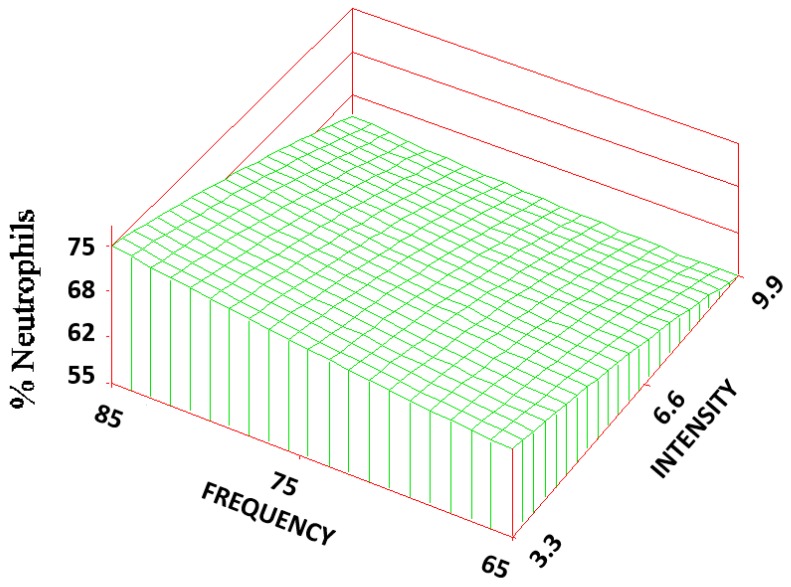
Surface response regarding the volume fraction of neutrophils. The regression equation is: N_V_ = 6.03 − 0.31(F) + 0.46(I) − 0.01(F)(I) + 0.3(I^2^).

**Figure 6:**
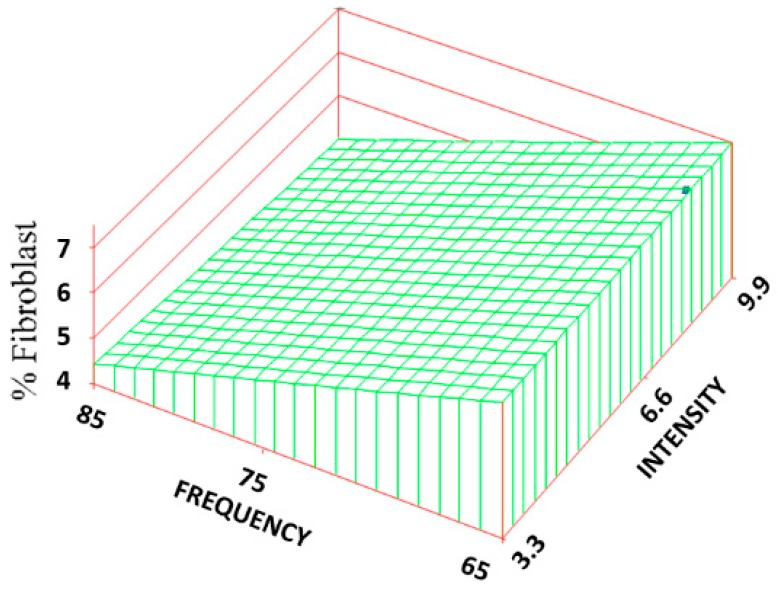
Surface response regarding the volume fraction of fibroblasts. The regression equation is: F_V_ = 4.57 − 0.08(F) + 1.63(I) − 0.02(F)(I) + 0.01(I^2^).

**Figure 7:**
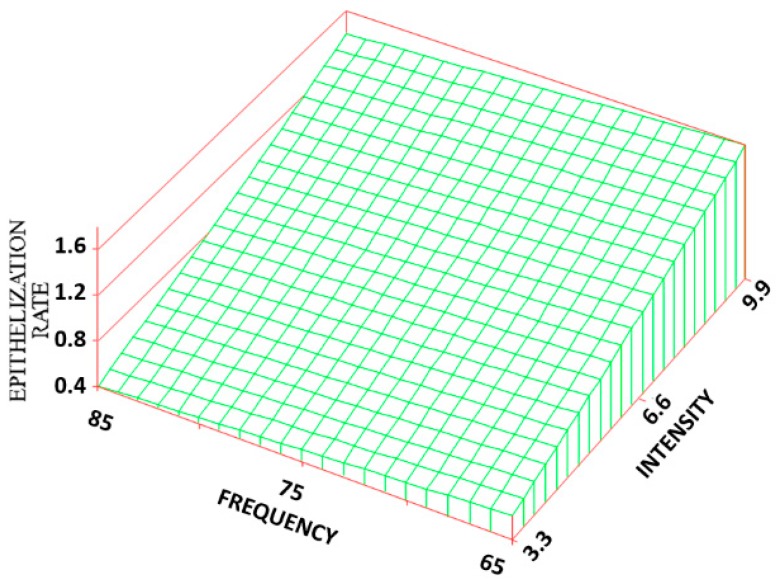
Surface response regarding the epithelialization rate. The regression equation is: ER = 0.34 − 0.11(F) + 1.18(I) − 0.01(F)(I) − 0.03(I^2^).

**Figure 8:**
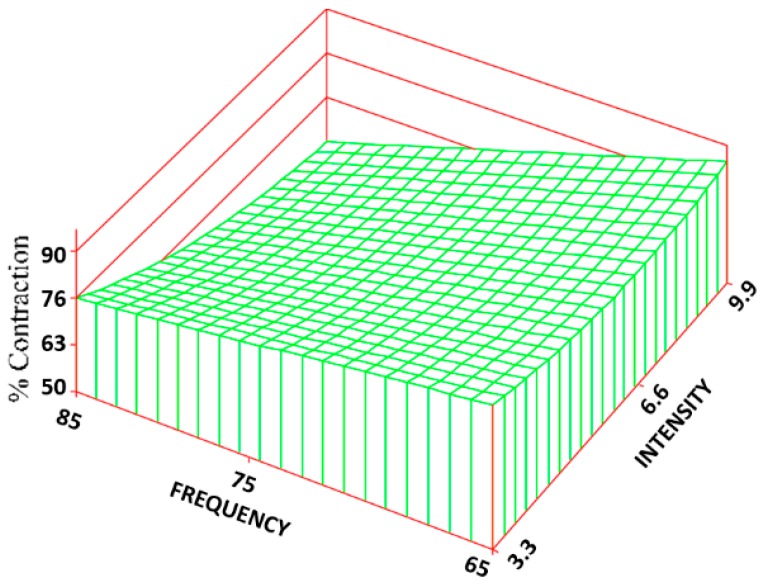
Surface response regarding the percentage of contraction. The regression equation is: CON = 89.9 − 0.21(F) + 6.81(I) + 0.01(F^2^) − 0.29(F)(I) + 0.82(I^2^).

**Figure 9:**
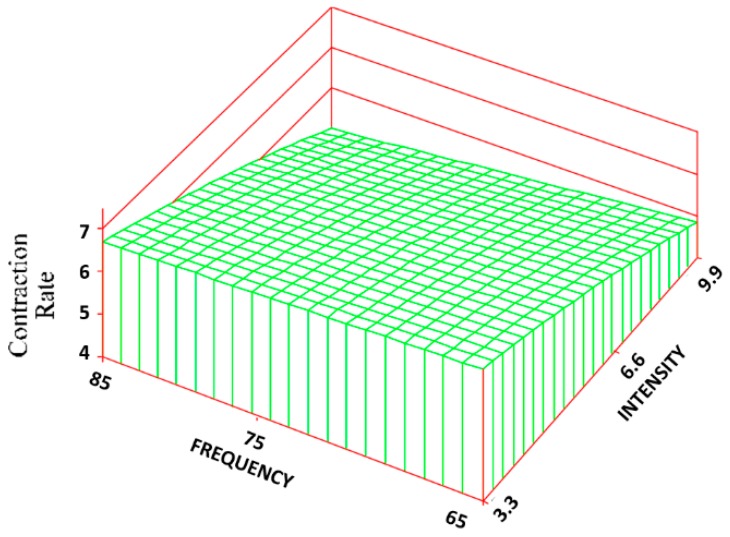
Surface response regarding the contraction rate. The regression equation is: CR = 6.73 − 0.01(F) + 0.49(I) − 0.02(F)(I) + 0.06(I^2^).

**Figure 10:**
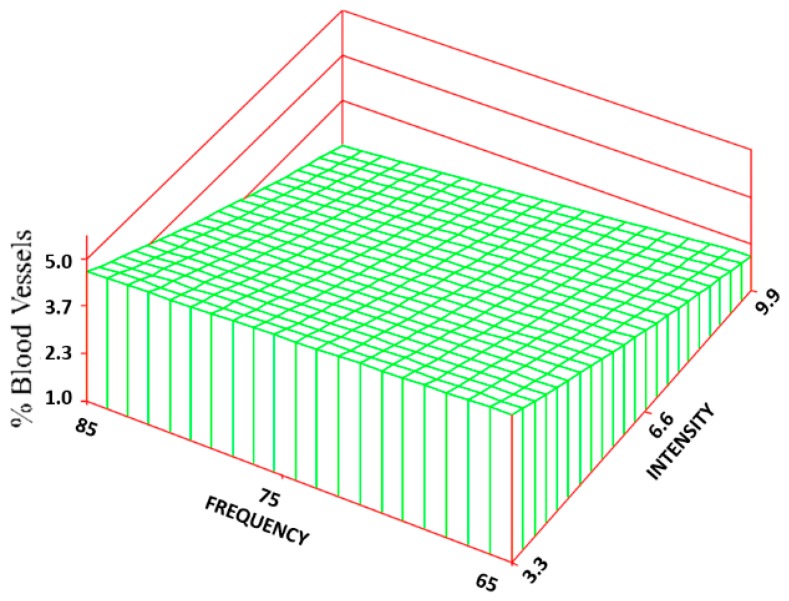
Surface response regarding the percentage of blood vessels. The regression equation is: BV = 0.89 − 0.84(I) + 0.01(F)(I) + 0.03(I^2^).

The histological results after the 2-week time period are summarized in Table 1 and Table 2. Utilizing the seven combinations of frequencies and intensities and each evaluated parameter response, a significant regression (*p* < 0.05) was produced. The resulting graphs, along with their corresponding regression equations, are shown in Figure 3, Figure 4, Figure 5, Figure 6, Figure 7, Figure 8, Figure 9 and Figure 10.

The ultimate tensile strength (TS) was found to vary significantly among the treatment groups (Figure 3). All the treatment groups were found to be significantly higher than the control group. The TS of the treated group ranged from 690 ± 228 to 1400 ± 404 KPa, which resulted in an increase of 28% to 160% over the control (537 ± 59 KPa), but it is still an order of magnitude lower than the reported strength of skin [36]. Based on the response surface regression (RSREG), the highest TS occurred at the lowest intensity level (3.3 Gauss), with TS equally proportional to intensity and frequency. The R^2^ value, which estimates the portion of the variation in the data that can be attributed to the model and not random error, was 0.45. This means that 45% of the variation in the TS data can be explained by the predicted regression model.

For the volume fraction of collagen (C_v_), it was found that at the lower intensity (3.3 Gauss) levels, there was a significant increase in C_v_ of the treatment group (maximum was 72% ± 7%) compared to that of the control group (62% ± 7%), approaching the normal skin level (75% ± 3%). At the highest intensity level (9.9 Gauss), however, there was little difference in C_v_ between the treatment groups and the control group. The RSREG (Figure 4) showed the collagen decreasing with increasing intensity levels, with changes in intensity having about twice the influence of changes in frequency. The R^2^ value for this regression was 0.26.

The volume fraction of the neutrophils (N_v_) of the three treatment groups (65, 6.6; 70, 3.3; and 85, 6.6) were statistically significantly lower (1/3–1/2 of the mean) than that of the control (6 ± 3%). The RSREG (Figure 5) showed that, the amount of neutrophils decreased as the frequency was lowered, with changes in frequency having about 10 times more influence than changes in intensity. The R^2^ value for this regression was 0.02. 

There was a statistically significant reduction in the volume fraction (1/4–1/3) of macrophages (M_v_) found between the treatment (all but the group (80, 3.3) and control groups that varied in a range from 4 ± 2% to 6 ± 2%. The RSREG of the macrophages showed a similar trend to that of the neutrophils but to a lesser degree.

Statistically significant increases (about 1/3 of the mean) were found between the volume fraction of fibroblast (F_v_) for two of the treatment groups (70, 3.3 and 70, 9.9) and the control group. The RSREG (Figure 6) showed the fibroblast level increased as both the frequency and intensity decreased, with changes in intensity having about twice the influence as changes in frequency. The R^2^ value for this regression was 0.04.

Although the ER rate of the treatment groups had no significant differences among themselves, with the maximum being 1.7 ± 0.8 mm/week, most (all but 80, 3.3) were significantly greater than that of the control group (a 75%–425% increase), which had the lowest ER rate (0.35 mm/week). The RSREG (Figure 7) demonstrated that the ER rate increased with increasing intensity, with both intensity and frequency changes having equal influence. The R^2^ value for this regression was 0.30.

There was little significant difference among the groups for the EPI. The EPIs in two treated groups were statistically different, which corresponded to 69.0 ± 26.6% for the treated group (70, 9.9) and 24.5 ± 12.7% for the treated group (80, 9.9), with no significant difference from that of the control observed.

The same frequency and intensity combinations were statistically different for both the CON (Figure 8) and CR rate (Figure 9). The control group exhibited the greatest amount of CON and CR rate (90% ± 6% and 7 ± 0.4 mm/week) with an approximate 53% and 80% increase over the treatment groups. Changes in intensity, however, had about 5 times the influence as changes in frequency. The R^2^ values for the CON and CR rate were 0.68 and 0.69, respectively.

For the WCR, no significant differences among the groups were found, but the control group healed mostly by contraction (94% of the healing) versus an average of 84% of the healing for the other groups. This corresponded to a contraction rate to epithelialization rate ratio of 16.8 for the control vs. an average of 6.5 for the treated groups (4.8 without the 80, 3.3 group).

Statistically significant increases (300%–584%) were found for the percentage of BV between most of the treatment groups (5.2 ± 4.7%—maximum value) and the control group (0.9 ± 1.3%). Based on the RSREG (Figure 10), the BV increased as the intensity increased, with little to no effect of changes in frequency. The R^2^ for this regression was 0.20.

## 4. Discussion

Designing degradable regenerative systems to optimize skin healing helps to understand the rates of migration of the key cells, since fibroblasts need to be within 100 μm of the blood supply to have sufficient oxygen and nutrients to survive and produce collagen. The needed rates for cell and tissue ingrowth vary based on the scaffold used. Both the chemistry and structure of the scaffold determine how much of the collagen scaffolding function can be taken up by the biomaterial and for how long based on the degradation rate. The scaffold and any added bioactivity can also alter the ingrowth rates. The study was designed to look at how electrical stimulation using PEMF alone can alter the rates. How the PEMF works in combination with a specific scaffold system would have to be evaluated, since there should be some synergistic relationships.

Although all the bioprocess rates are important, the study was designed to just look at the clinically important rates (ER rate and CR rate) and just determine the amount of the cells and tissue present (essentially the bioprocesses that determine the healing rate) in the wound bed. There are some data on how these bioprocesses occur in wounds with and without scaffolds. Fibroblasts migrate up to 200 μm/day [47,48], but will only do so if the BV and nutrients are within 100 μm. Angiogenesis for a 0.5 mm collagen/GAG artificial skin system takes 7–9 days [47,48] (50–70 μm/day). This slows the fibroblast ingrowth across 500 μm for 5–8 days (60–100 μm/day) versus 2.5 days (200 μm/day). 

Therefore, angiogenesis becomes the rate-limiting step. Angiogenesis is also important to support the epidermal layer. Epidermis migrates at about 2.3 mm week over viable vascularized dermis, but only half that if it has to burrow through tissue to find viable tissue [49]. Therefore, the speed in which vascularized connective tissue moves into the wound determines both the speed and the earliest time point at which epidermal cells can begin to migrate, at least a week for the 500 μm collagen/GAG artificial skins.

Angiogenesis is also the key in reducing the risk of infection because it can bring cells for an acute inflammatory response to kill bacteria [49]. Researchers have tried to circumvent the slow angiogenesis by providing a cell culture like medium for the cells (with antibiotics) [50]. This may not be logistically possible or practical in all cases.

Therefore, strategies to speed up angiogenesis are critical in the early stages of healing. This requires not only stimulation of vascular ingrowth, but also fibroblast and tissue ingrowth, to provide the collagen scaffold for the vascular tree. A functional biomaterial scaffold would reduce the need for fibroblasts to produce the collagen scaffold, but this study wanted to look at the ability of PEMF alone.

Again, clinically for a degradable regenerative system, it is the rate of wound closure primarily by the ER rate that is important. Early angiogenesis is necessary for ER to occur in the latter stages [13]. Electrical stimulation is one technique that has shown the ability to speed all the necessary steps for skin wound healing and therefore speed overall healing. It is also beneficial to speed healing without the need for added bioactivity (i.e., cells or growth factors) as well as be done at the patient’s home to reduce both development cost and cost of the treatment. Further, the potential to tailor the treatment to the application, stage of healing, and needs of a specific case makes electrical stimulation an attractive treatment option.

As previously mentioned, the benefit of electrical stimulation can be that it both mimics the normal “current of injury” when it is absent as well as amplifying it when it is present, stimulating a non-healing or chronic wound to heal as well as speeding the healing in an acute wound [20,21,22,23,24,25]. Numerous studies have been done on the electric fields in animals that regenerate [51,52]. It has been shown that changes in the field can prevent limb regeneration. The field has been measured at 100 μA/cm^2^ [52]. It actually is a relatively constant voltage with a changing current that is dependent on the tissue resistance, which changes as the tissue heals [53]. Although insight can be obtained from limb regeneration, it is more complicated than just a skin wound, since bone is also involved [53,54].

For dermal scaffolds, it is therefore important to understand what electric fields do to cells in soft tissue. Electrical stimulation has shown promise in speeding up healing by activation of the key cells. Specifically for fibroblasts, electrical fields have been shown to significantly increase the synthesis rates of both proteins (including collagen) and DNA [55] as well as the expression of receptors for transforming growth factor-β [18]. Further, the electric fields have been shown to increase motility [56] with cells oriented perpendicular to the field lines. Similarly for keratinocytes, electrical fields have stimulated more advanced differentiation [57,58] and calcium-dependent cathodic migration [59]. Additionally, it has been shown that electrical stimulation with positive polarity can decrease mast cells, leading to decreased scar thickness and a better cosmetic appearance in treated wounds [60].

In an in vitro study, keratinocytes and fibroblasts were exposed to different levels of voltage (around the levels seen in regenerating animals) and were checked for up- or down-regulation of gene expression [53,54]. It had been anticipated that specific changes would be found for each type of field. Instead, each field altered multiple pathways that would have both positive and negative impact on healing [53,54].

PEMF has the ability to stimulate healing similar to other electrical stimulation systems, but does not require surgery or contact with the wound. Numerous investigators have used PEMF in animal models and clinically. Ottani et al. exposed rats to a PEMF (50 Hz, 8 mT peak) every 12 h. They concluded that the vascular network formed early and enhanced the development of cellular organization, collagen formation and maturation [40]. Greenough examined the effects of PEMF on angiogenesis in the rabbit ear model. Using various waveforms at a frequency of 72 Hz, he found a significant increase in the growth and formation of vascular tissue over the controls [42]. Murray et al. found that fibroblast activity increased in vitro, using a 15 Hz PEMF. They concluded that PEMFs can increase the net production of collagen due to a decrease in intracellular degradation of synthesized collagen, possibly through a modification of cAMP metabolism [43]. 

Clinically, in a study by Ieran et al., 44 patients with venous ulcers were randomly placed in either a placebo group or a PEMF treatment group. It was concluded that the PEMF (2.8 mT field at a frequency of 75 Hz with an impulse width of 1.3 msec) resulted in an increase in the healing rate by 66.6% with a recurrence in only 25% of the cases [38]. A Diapulse^®^ machine [37] using 27 MHz modulated with a 65 µsec pulse at 80–600 Hz was found to double the amount of wounds healed after seven days. Others have also seen increases in healing rates for venous ulcers [39] as well as increases in rates of wound CR, collagen formation, and neo-angiogenesis in a rat model [40]. 

Though several studies have reported the benefits of using PEMF to accelerate the healing response in soft tissue wounds, each study has used a different combination of frequency and intensity levels. Determining the relationship between these parameters and soft tissue healing is needed to determine if there is an optimal PEMF regime that could be used clinically.

The data presented in this study suggest that altering the combination of frequency and intensity levels of PEMF produces different effects on the wound healing process. Not only were there differences among the treatment groups, but there was an overall enhancement of the healing response between each treatment group and the control group. These results paralleled the previous study [44], which looked at only the middle combination, but at both one and two weeks. 

By altering the intensity levels of the field, the TS, volume fraction of collagen, ER rate, CR rate, CON, and volume fraction of BV were altered. Both the TS and collagen were greatest at the lower intensity (3.3 Gauss) and then began to decrease as the intensity was increased. This may be partly a result of PEMF effect on the CR rate. At the low intensities, with the CR rate being the highest, a more densely packed collagen and thus a higher wound TS would be generated. As the CR rate decreased with increasing intensity, the volume faction of collagen and TS also began to decrease. Interestingly, however, the control group had the highest CR rate but the lowest TS and volume faction of collagen. 

The results suggest that more collagen was produced in the treatment groups than that in the control group. The ER rate, however, increased with increasing intensities, resulting in an insignificant change in the overall WCR (CR rate plus ER rate). The control group, however, healed much more by contraction than all but one of the treated groups (80, 3.3). The PEMFs were able to increase the ER rate to close to the 2–3 mm/week reported over moist healthy tissue [49]. This probably works by reducing the lag phase where no ER occurs due to the lack of a regenerated healthy dermis. Assuming a constant ER rate (2.33 mm/week), once the dermis healed, the control (0.35 mm/week) would correspond to a 11.9 ± 1.2 day lag while the groups (70, 9.9; 85, 6.6) (1.7 mm/week) would correspond to only a 3.8 ± 5.4 day lag—a reduction of about one week in the lag phase. Similarly in a previous study [58], the control group and treatment groups for one-week treatment exhibited a lag phase of 5 days and 2 days, respectively

The absence of a significant change in the EPI among the various groups was probably due to the inverse relationship between the CR rate and ER rate. As the CR rate decreased, there was more wound area to be epithelialized, but the ER rate was increasing. The rate measures are a better indication of how well the wound is healing because the percentage measures are dependent on wound size. The BV exhibited the same trend as the ER rate: increased angiogenesis with increased intensity levels. 

The change in collagen was similar to changes in fibroblast ingrowth, with both having changes in the intensity level had about twice the influence of changes in frequency, and collagen being slightly more influenced by intensity than fibroblasts. The regression equation for collagen also was a more accurate model for the data than the regression equation for fibroblasts. It has been suggested that fibroblasts, under the influence of PEMF, increase in size, and accelerate protein synthesis, but do not undergo cell division [15,43]. Therefore, increases in collagen density do not need to be accompanied by an increase in the number of fibroblasts. The collagen density should be a function of the CR rate, amount of angiogenesis as well as number of fibroblasts. In this case, however, it appears that the CR rate had the most influence, followed by the amount of fibroblasts, and then the amount of BV. 

The frequency of the field appeared to have the most influence on the inflammatory cells: neutrophil and macrophage volume fractions. As the frequency increased, the volume fraction of the inflammatory cells increased, reaching a plateau (75–80 Hz) close to the control value, and then decreased. The intensity level, in these cases, did not seem to significantly influence the cellular response. It was also observed upon histological evaluation; cells in the treatment groups were characterized by a more intense stain than those in the control group, suggesting a change in the cellular activity.

A consideration with this study is that only one time period was evaluated. In previous studies, with wounds similar to this one, it was determined that healing was nearly completed by 2 weeks in the treatment groups and by 3 weeks in the control group [44,61,62,63,64]. The previous study [44] showed similar trends to this study with significant increases in collagen and fibroblast volume fractions at both one and two weeks, significant decreases in macrophages at both one week and two weeks with neutrophils only and one week, and significant increases in angiogenesis at week one. It was also seen that ER had just started by one week (0.7 mm/week for the control and 1.7 mm/week for the treated group). Since similar trends were seen at one and two weeks and healing was further along, but not complete, 2 weeks was selected as a good time to compare treatments for this study. 

Another concern is that multiple wounds were used from the same animal. Previous studies [44] have indicated that although there is a high animal-to-animal variability, the wound-to-wound variability, within the same animal, is comparable as long as there are not systemic problems with the animals. It appeared to be similar in this case with a similar variability among wounds within the same animal to those between animals. 

There is also a concern that the statistical differences seen do not necessarily mean a clinically significant difference. The study was designed, with an n of 8 for each test, in that the change in means between treatment and control needed to be at least equal to the standard deviation to be statistically significant. The precision of each measure, standard deviation as a percentage of the mean (2.8 times the coefficient of variation in this case), varied among the variables tested with the lowest volume fraction of collagen, and TS, CON, CR rate, and WCR all below 11% for the controls. The percentage of BV was the highest (144% for the control) with the other measures around 50% for the standard deviation divided by the mean. Therefore, the sensitivity of the study to determine statistical significance was different among the parameters and was affected by the precision of the measurement.

It is difficult, however, to even know what changes in histological parameters are clinically relevant. The 3–6-fold increase in BV, as well as the 1/3 decrease in inflammatory cells and 1/3 increase in fibroblasts between controls and treatments, should probably be a clinically significant difference. Mostly, however, these differences help to understand why the changes in outcome measures occur (healing rates and TS). Again, PEMF is intended to be used with other modalities, particularly degradable regenerative scaffolds, to enhance their regenerative ability. Different applications would have different design constraints as well as what level of improvement would be clinically significant. In some applications, the scaffold alone can be sufficient, and in others, it would need some adjunctive therapy. Using electrical stimulation in most cases is more desirable than added biologics, if it can help push the system past the minimum design constraints.

For example, electrical stimulation used alone on pressure ulcer patients provided a clinically significant increase in the healing rate, but did not meet the doubling of the rate needed over the entire healing cycle [13,65]. Additionally, fibrin glue alone has shown to lead to a clinically significant difference, when used to adhere skin grafts, in burn patients with added growth factors not making a significant clinical improvement to be justified [66]. 

The 28%–160% increase in TS should be clinically significant as well as the 75%–425% increase in the ER rate and the 50%–80% decrease in the CR rate. This increase in the ER rate would correlate to a reduction in healing time, that is, healing in 1/2 to 1/4 of the time, assuming the same amount of CR. In addition, the actual effect on ER and CR clinically needs to be determined. The model is somewhat predictive [65], but rabbits typically heal more by contraction than would be seen clinically. Also the presence of a scaffold greatly reduces the contraction rate; by over 85% in a similar model in another study in this issue. If the contraction rate is reduced by 85% then one of the treatments (85, 6.6) would reach the goal of doubling the healing rate and another would be close (70, 9.9).

The overall WCR did not significantly change, in this study, due mostly to the high CR. The increases seen in ER should reduce the scarring. The reduction in the CR/ER ratio seen should significantly affect the quality of the tissue repair, leading to stronger tissue with less scarring. Although the increase in the ER rate has the potential to meet the doubling of the healing rate design constraint, the study only looked at one time point and some clinical studies have shown this increase is only sustainable for 3 weeks [13,65].

Clinical significance is also related to the type of healing. Is a shorter healing time, which may include scarring and significant amounts of CR, more advantageous than a slower healing time that involves a more regenerative response? This is also application-dependent. For instance, in pressure ulcers, the most important factors are a reduction in the healing time and in the chance of recurrence. In burns, a limited CR and reduction in scarring are more important. The study seems to indicate that by changing variables of the PEMF, it is possible to tailor the response to the application.

The regression equations can help in predicting the values of the parameters for frequency and intensity combinations which were not tested. Standard deviations higher than 50% of the mean probably served to reduce the R^2^ values for the volume fractions of cells (about 0.03) as well as reducing the value for the volume fraction of BV (0.2). The models were most predictive for CR (about 0.65) and TS (0.45) with a reasonable predictability for the ER rate (0.3) and percentage of collagen (0.26). The models will also be helpful when the clinical data with a specific scaffold is obtained, to allow selection of likely PEMF treatments. 

These differences seen among treatments, in this study, may help explain why some PEMF treatments have led to accelerating wound healing while other studies showed little differences between the PEMF and control groups [37,38,39,40,41,42,43]. In general, the study showed that a PEMF can speed up the healing process probably by shortening the inflammatory phase. Specifically, a reduction in inflammatory cells was seen at two weeks (one week in the previous study [44]) as well as more of the repair phase indicated by more BV, more fibroblasts, and increased epithelial healing with less CR. These enhancements in healing are similar to those found in previous studies using electrical stimulation. These studies have shown how it is chemotactic for inflammatory cells [27], activates fibroblasts to increase collagen production [16,40,43,44,45,46,47,48,49,50,51,52,53,54,55] and enhance proliferation [17,19,44,56], enhances ER [54] and overall healing rates [20,22,23,24,25,26,27,28,34,38], reduces CR and scarring [61], and enhances angiogenesis [34,40,42,44].

This data also support the frequency and field strength “windows” that have been reported in the healing of non-unions of bone [67]. The “window” effect means that there are specific PEMFs that accelerate the healing of bone, while others do not. It is therefore reasonable to expect that soft tissue healing would behave similarly, with optimal wound healing occurring only in specific “windows” of exposure. Again, in one clinical study, with one type of field, the benefits only lasted 3 weeks [65].

The reason for choosing this time window for electrical stimulation may be due to effects at the cellular level. It has been suggested that breakdown of cell membranes for electroporation is the product of voltage and time [68]. With electroporation using kV for milliseconds, it reasonable to expect cell changes using μV for millions of seconds.

The study also indicates that an optimum treatment may require different fields at different stages of the healing process, even when using a regenerative scaffold. For example, during the early stages of healing, you could use a system with low intensity and low frequency to more quickly resolve the inflammatory phase, reducing the number of inflammatory cells. Then, the system could be switched to a system with high intensity and low frequency to stimulate granulation tissue formation by increasing angiogenesis and fibroblast ingrowth. Once the system granulates, this same system can be used to stimulate an ER rate while maintaining a low CR rate. Once the system heals, it could be switched to a low-intensity system to stimulate fibroblasts to remodel the collagen and increase wound strength.

In summary, it was shown that PEMFs at different combinations of frequency and intensity levels can be used to enhance overall healing of full-thickness defects in a rabbit model. Additionally, the study suggests that by changing the frequency and intensity levels of the PEMF, TS, amount of collagen, CR rate, and ER rate, and angiogenesis could be altered. It is possible that an optimal PEMF system involves a series of different frequency and intensity levels at various stages of the healing process. Further studies are needed to both further optimize the system for specific clinical applications and to further elucidate the mechanism of healing enhancement by electrical stimulation. It is also possible to use the PEMF as an adjunctive therapy (possibly to replace added biologics) to enhance the regenerative ability of a degradable scaffold; but the synergistic effect of PEMF is likely to be different for different scaffold systems.

## Figures and Tables

**Table 1:** Tensile strength (TS) and histomorphometry.

Groups Freq, Int	Collagen (%)	σ_uts_ (KPa)	Neutrophil (%)	Marophage (%)	Blood Vessel (%)	Fibroblast (%)
CONTROL	61.7 ± 6.6	537 ± 59.3 ^c^	6.1 ± 2.6	5.8 ± 2.0 ^b^	0.9 ± 1.3 ^c^	4.6 ± 1.9
65, 6.6	70.6 ± 6.2 ^a^	690 ± 228 ^a^	3.8 ± 2.6 ^a^	4.2 ± 1.9 ^a^	1.7 ± 2.2	4.8 ± 2.4
70, 3.3	72.4 ± 6.9 ^a,b^	1410 ± 373 ^a^	3.5 ± 1.5 ^a,c^	4.1 ± 2.2 ^a^	1.0 ± 1.6	6.4 ± 3.0 ^a^
70, 9.9	62.1 ± 9.2	827 ± 232 ^a^	4.9 ± 2.3	3.8 ± 2.4 ^a,c^	4.1 ± 4.1 ^a^	6.6 ± 2.9 ^a,b^
75, 6.6	61.3 ± 8.0	752 ± 415 ^a^	7.3 ± 6.2 ^b^	4.6 ± 3.0 ^a^	2.6 ± 2.7 ^a^	6.0 ± 3.2
80, 3.3	72.1 ± 5.3 ^a,b^	1400 ± 404 ^a,b^	6.5 ± 3.1	5.3 ± 2.5	1.3 ± 1.7	5.4 ± 2.7

^a^, significantly different from control at *p* < 0.05; ^b^, maximum value; ^c^, minimum value.

**Table 2:** Contraction and epithelialization.

Groups Freq, Int	Percentage of Epithelialization (%)	Epithelialization Rate (mm/week)	Percentage of Contraction (%)	Contraction Rate (mm/week)	Wound Closure Rate (mm/week)
CONTROL	56.4 ± 34.3	0.35 ± 0.2 ^c^	89.7 ± 6.0 ^b^	6.7 ± 0.4 ^b^	7.1 ± 0.4
65, 6.6	48.4 ± 13.6	0.8 ± 0.2 ^a^	77.9 ± 3.1 ^a^	5.8 ± 0.2 ^a^	6.6 ± 0.3
70, 3.3	59.5 ± 24.3	0.7 ± 0.3 ^a^	85.3 ± 3.5	6.4 ± 0.3	7.1 ± 0.3
70, 9.9	69.0 ± 26.6 ^b^	1.7 ± 1.0 ^ab^	63.2 ± 18.4 ^a^	4.7 ± 1.4 ^a^	6.5 ± 0.9
75, 6.6	39.6 ± 31.8 ^a^	1.4 ± 1.0 ^a^	51.3 ± 3.7 ^a,c^	3.9 ± 0.3 ^a,b^	5.3 ± 1.2
80, 3.3	48.2 ± 27.6	0.4 ± 0.2	87.8 ± 5.1	6.6 ± 0.4	7.0 ± 0.3
80, 9.9	24.5 ± 11.7 ^a,c^	0.9 ± 0.3 ^a^	52.4 ± 7.7 ^a^	3.9 ± 0.6 ^a^	4.8 ± 0.8 ^c^
85, 6.6	60.3 ± 27.3	1.7 ± 0.8 ^a,b^	58.9 ± 13.8 ^a^	4.4 ± 1.0 ^a^	6.5 ± 0.7 ^b^

^a^, significantly different from control at *p* < 0.05; ^b^, maximum value; ^c^, minimum value.
